# Prevalence of Multidrug-Resistant Gram-Negative Bacilli Causing Neonatal Sepsis: A Retrospective Study in a Tertiary Care Hospital From Eastern India

**DOI:** 10.7759/cureus.93476

**Published:** 2025-09-29

**Authors:** Rajesh K Dash, Santosh K Panda, Shradha Smriti, Swarupa Mohapatra, Ipsa Mohapatra, Nipa Singh, Dipti Pattnaik, Sushree S Behura, Soumini Rath, Manas K Nayak, Subhra Snigdha Panda

**Affiliations:** 1 Department of Microbiology, Kalinga Institute of Medical Sciences, Bhubaneswar, IND; 2 Department of Pediatrics, Kalinga Institute of Medical Sciences, Bhubaneswar, IND; 3 Department of Community Medicine, Kalinga Institute of Medical Sciences, Bhubaneswar, IND

**Keywords:** amr, mdr gnb, neonatal sepsis, prevalence of gnb, risk factors

## Abstract

Background: Neonatal sepsis is considered one of the major causes of morbidity and mortality in low and middle-income countries, with a rising burden of multidrug-resistant (MDR) gram-negative bacilli (GNB). The emergence of antimicrobial resistance (AMR) has significantly compromised treatment outcomes in neonatal intensive care units (NICUs). This study aimed to determine the prevalence, antibacterial susceptibility patterns, and associated risk factors of MDR GNB among septic neonates over a four-year period.

Materials and methods: A retrospective study was carried out from January 2021 to December 2024 in a tertiary care health setting in India. Data regarding septic neonates were retrieved from NICU records, and culture details were retrieved from the LIS (laboratory information system). Blood and cerebrospinal fluid (CSF) samples from neonates with clinically suspected sepsis were processed using the BACT/ALERT® three-dimensional (3D) system (bioMérieux SA, Marcy-l'Étoile, France). Identification and antibiotic sensitivity testing were done by the VITEK® 2 Compact automated system (bioMérieux SA), and interpretation was done as per standard microbiological protocol. Demographic, clinical, and bacterial profile data were analysed using the IBM SPSS Statistics for Windows, version 27.0 (IBM Corp., Armonk, New York, United States)

Results: Altogether 2,670 neonates were admitted to the NICU during the study period. Out of 424 culture-positive samples, 201 (47.4%) were gram-negative isolates. The most prevalent bacteria were *Klebsiella pneumoniae* (26.4%), followed by *Acinetobacter spp.* (23.9%), and *Escherichia coli* (10.4%). The highest number of MDR strains was observed in *K. pneumoniae *isolates (73.6%), followed by *E. coli* (61.9%). *K. pneumoniae* showed better sensitivity to trimethoprim/sulfamethoxazole (57.64%) and gentamicin (53.13%), *Acinetobacter spp.* to minocycline (77.4%) and ciprofloxacin (66.7%), and *E. coli* to gentamicin (60%) and carbapenems (50%). Neonates with low birth weight (LBW) and very low birth weight (VLBW) showed a higher proportion of MDR infections than extremely low-birth-weight neonates (ELBW) (p= 0.02). These observations emphasize the importance of implementing antimicrobial surveillance and tailored antibiotic strategies in managing neonatal sepsis.

Conclusion: The findings highlight the predominance of MDR GNB in neonatal sepsis and emphasize the need for species-specific antibiotic strategies with continuous resistance monitoring.

## Introduction

Neonatal sepsis is a systemic pathological condition that occurs within 28 days of age after birth involving pathogenic microorganisms. It results in various hemodynamic changes and clinical manifestations. In developing nations, neonatal sepsis accounts for about 36% of all neonatal deaths and may go up to one million deaths annually [1-3]. Depending upon onset, neonatal sepsis is classified as early-onset sepsis (EOS) occurring in less than 72 hours after birth and late-onset sepsis (LOS) when it manifests beyond 72 hours of life up to 28 days [4].

The bacteria causing neonatal sepsis vary across regions and even within hospitals. In developing countries, bacteria like *Klebsiella pneumoniae*, *Staphylococcus aureus*, and coagulase-negative *Staphylococcus *(CONS) are found as predominant microorganisms causing neonatal sepsis, often showing resistance to first-line antibiotics [5]. Neonatal sepsis cases due to gram-negative bacilli (GNB) are considered to be an increasing global burden, with the rise of multidrug resistance, which poses major challenges to effective treatment and outcomes [6]. Multidrug-resistant (MDR) bacteria show acquired resistance to a minimum of one antimicrobial agent in three or more different classes of antibiotics [7]. Globally, GNB account for nearly 60% of neonatal sepsis cases. In India, GNB are also the major culprits, with studies reporting a high frequency of multidrug resistance among isolates [8,9]. MDR *Enterobacteriaceae* constitute over 80% of GNB isolates, with carbapenem-resistant strains emerging as a major therapeutic challenge [10].

Antimicrobial resistance (AMR) has become a critical issue in healthcare settings that accounts for approximately 31.0% of neonatal sepsis-related deaths [11]. A comprehensive understanding of the predominant bacterial pathogens and their antibiotic susceptibility patterns is crucial for guiding effective empirical therapy [12]. The growing antibiotic resistance, coupled with the limited development of new antibiotic agents, again exacerbates the complexity of managing neonatal sepsis [13].

Neonatal sepsis predominantly affects newborns who are preterm and of low birth weight because of their underdeveloped immunity and physiological barriers [14]. This is further influenced by several risk factors such as preterm birth, prolonged rupture of membranes, maternal infections, and prolonged hospitalization. Neonates belonging to low socio-economic groups or rural backgrounds are more susceptible to septicemia because of their greater exposure to unhygienic environments [15].

This study is planned with an aim to find out the prevalence of MDR GNB strains causing neonatal sepsis, analyse the antibacterial susceptibility patterns, and evaluate the associated risk factors over a four-year study period.

## Materials and methods

Operational definition

The term neonatal sepsis is characterized by a systemic inflammatory condition caused by the invasion of microbial agents into sterile body fluid compartments like blood or CSF [16]. Neonatal sepsis includes both blood culture-positive sepsis and clinical sepsis. Clinical sepsis is defined as a condition in neonates with clinical signs and symptoms of sepsis, like lethargy or irritability, poor feeding, feed intolerance, respiratory distress, thermal instability, seizure, shock, along with the presence of supportive biomarkers such as C-reactive protein (CRP) or procalcitonin (PCT). Culture-positive sepsis is confirmed by isolation of a microorganism from blood culture in neonates with the presence of clinical features of sepsis [17].

Study setting, data gathering, and study duration

This was a retrospective study conducted over four years, from January 1, 2021, to December 31, 2024, in the Department of Microbiology in association with the NICU at Kalinga Institute of Medical Sciences, Bhubaneswar, Odisha, India. This hospital has a state-of-the-art NICU with a total capacity of 40 beds. The NICU has the capability of managing a substantial annual influx of neonates requiring critical care. The study was approved by the Institutional Ethics Committee, Kalinga Institute of Medical Sciences, Kalinga Institute of Industrial Technology University (approval number: KIIT/KIMS/IEC/1982/2025).

Relevant data regarding septic neonates were collected from NICU records. Blood culture data were retrieved from the Laboratory Information System (LIS) for all samples included during the study period. The variables extracted included patient age, sex, gestational age, mode of delivery, place of birth, outcome, bacterial species, year of isolation, and the antibiotic susceptibility profiles of the isolates. Neonates are classified according to gestational age as preterm (<37 weeks), term (37-42 weeks), and post-term (>42 weeks). Based on birth weight, they were further classified as LBW (1.5-2.5 kg), VLBW (1.0-1.5 kg), and ELBW (<1.0 kg) [18].To ensure data accuracy, repetitive isolates from the same patient were excluded, and only the first isolate was included in the analysis.

Sample collection and processing techniques

Blood and CSF samples (wherever required) from neonates with clinical signs and symptoms of sepsis were cultured by the automated blood culture system (BACT/ALERT® three-dimensional (3D) system; bioMérieux SA, Marcy-l'Étoile, France). A volume of 1.5 -2 mL of blood (0.5-1 mL CSF, where applicable) under aseptic conditions, prior to antibiotic administration, into BacT/ALERT PF Plus bottles (bioMérieux SA) (specific for paediatric patients). As per the available data, a single blood culture sample drawn in a sufficient volume (with 1 ml of blood as the minimum volume) can be used for the diagnosis of neonatal sepsis [19]. A sample was incubated in the automated system for up to five days in case of a negative culture result or until positive growth was detected as per the manufacturer’s guidelines. Positive blood cultures were sub-cultured onto blood agar and MacConkey agar, with petri plates incubated for 24 hours at 37°C and monitored for bacterial growth. 

Cultures such as coagulase-negative *Staphylococci*, *Corynebacterium *spp., *Micrococcus* spp., and* Bacillus *spp., which were normal skin commensals and had no supportive clinical evidence, were excluded as contaminants from the study.

Bacteria identification and antibiotic sensitivity testing

Bacteria identification and antibiotic sensitivity testing were done by VITEK® 2 Compact automated system (bioMérieux SA). Antibiotic susceptibility results were interpreted as per Clinical and Laboratory Standards Institute (CLSI) guidelines [20]. In this study, MDR strains of bacteria were identified as the isolates exhibiting non-susceptibility to at least one antibiotic from three or more different classes of antibiotics [7]. The antibiotics on the VITEK 2 panel with their respective drug classes are as follows: ampicillin (β-lactam); amoxicillin/clavulanic acid and ticarcillin/clavulanic acid (β-lactam/β-lactamase inhibitor combinations); piperacillin/tazobactam (extended-spectrum penicillin/β-lactamase inhibitor); ceftazidime, ceftriaxone, cefepime (cephalosporins); cefoperazone/sulbactam (cephalosporins/β-lactamase inhibitor); aztreonam (monobactam); imipenem, meropenem, and ertapenem (carbapenems); amikacin and gentamicin (aminoglycosides); ciprofloxacin and levofloxacin (fluoroquinolones); minocycline (tetracycline); tigecycline (glycylcycline); and trimethoprim/sulfamethoxazole (folate pathway inhibitor).

Data analysis

The collected data were compiled using Microsoft Excel (Microsoft Corporation, Redmond, Washington, United States) and analysed using IBM SPSS Statistics for Windows, version 27.0 (IBM Corp., Armonk, New York, United States). Categorical variables were expressed as frequencies and percentages, while continuous variables were summarized as mean with standard deviation or median with interquartile range, as appropriate. Chi-square and Fisher’s exact tests were performed for statistical analysis, and a p-value less than 0.05 was taken to be statistically significant.

## Results

A total of 2,670 neonates, admitted in the NICU of the hospital from January 2021 to December 2024, were included in the study. During this period, 2,425 samples, including blood and CSF, were collected from the neonates with clinical features of sepsis and sent to the microbiology laboratory for culture analysis. Out of this, 424 samples obtained from 424 neonates showed positive culture growth. All 424 culture samples showed a single type of microorganism growth, resulting in the isolation of a total of 424 microorganisms. Among the total 424 isolates, 186 were identified as gram-positive bacilli, 201 as GNB, and 37 as *Candida* spp.. Among the 201 GNB isolates obtained from 201 neonates, 192 isolates were recovered from the blood samples of 192 neonates, and nine isolates were recovered from the CSF of nine neonates. The prevalence of GNB causing neonatal sepsis was calculated to be 47.40% (201/424). Figure 2 shows the entire sample segregation process.

**Figure 1:**
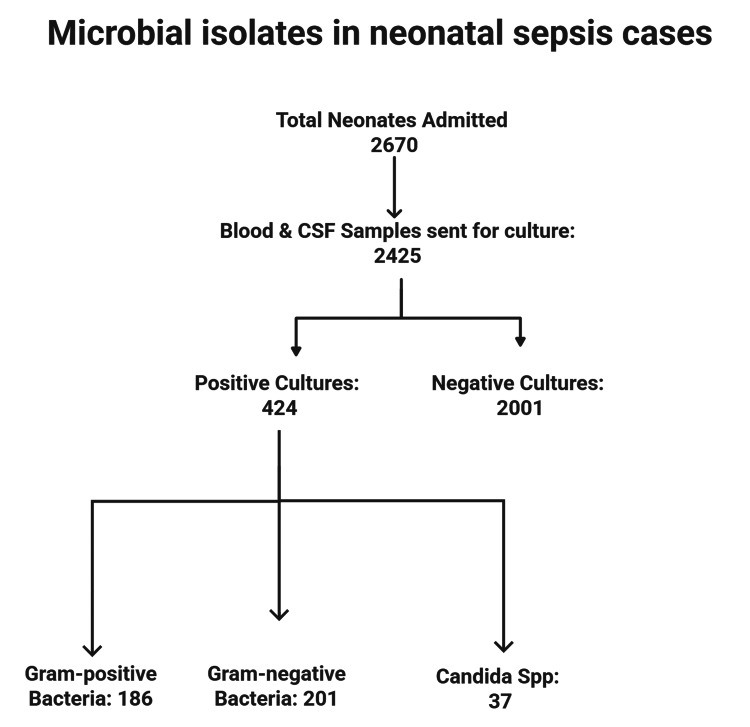
Flowchart showing sample segregation

Among 201 neonates, 122 (60.7%) were male and 79 (39.3%) were female, with the most being preterm neonates (n=151, 75.1%). There were 72 (35.8%) VLBW neonates, followed by 49 (24.4%) ELBW neonates, and 43 (21.4%) LBW neonates. A total of 106 (52.7%) were born through lower segment caesarean section (LSCS), with 107 (53.2%) being delivered in an outside institution, and the overall mortality rate was found to be 20.4% (41/201) (Table 1).

**Table 1:** Demographic and clinical characterization of the neonates (N = 201)

Variable	Category	Frequency (Percentage)
Gender	Male	122 (60.7%)
	Female	79 (39.3%)
Gestational Age	Preterm	151 (75.1%)
	Term	49 (24.4%)
	Post-term	1 (0.5%)
Birth Weight	Extremely low birth weight	49 (24.4%)
	Very low birth weight	72 (35.8%)
	Low birth weight	43 (21.4%)
	>2500 g	37 (18.4%)
Mode of Delivery	Lower segment caesarean section	106 (52.7%)
	Vaginal Delivery	95 (47.3%)
Place of Birth	Inborn	94 (46.8%)
	Outborn	107 (53.2%)
Outcome	Discharged	160 (79.6%)
	Death	41 (20.4%)

**Figure 2:**
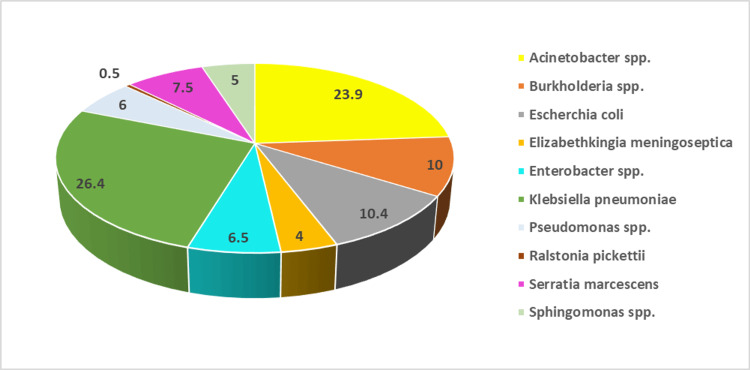
Distribution of gram-negative bacilli among total study isolates in percentage (N=201)

Among all the GNB isolates, *K. pneumoniae *was found to be the predominant bacterium with a prevalence of 26.4% (53/201), followed by *Acinetobacter* spp. (23.9%, 48/201) and *E. coli *(10.4%, 21/201). Other isolates included *Burkholderia* spp. (10.0%, 20/201), *Serratia marcescens* (7.5%, 15/201), *Enterobacter* spp. (6.5%, 13/201), *Pseudomonas* spp. (6.0%, 12/201), *Sphingomonas* spp. (5.0%, 10/201), and *Elizabethkingia meningoseptica* (4.0%, 8/201). Only one (0.5%) isolate of *Ralstonia pickettii *was obtained (Figure 2).

**Figure 3:**
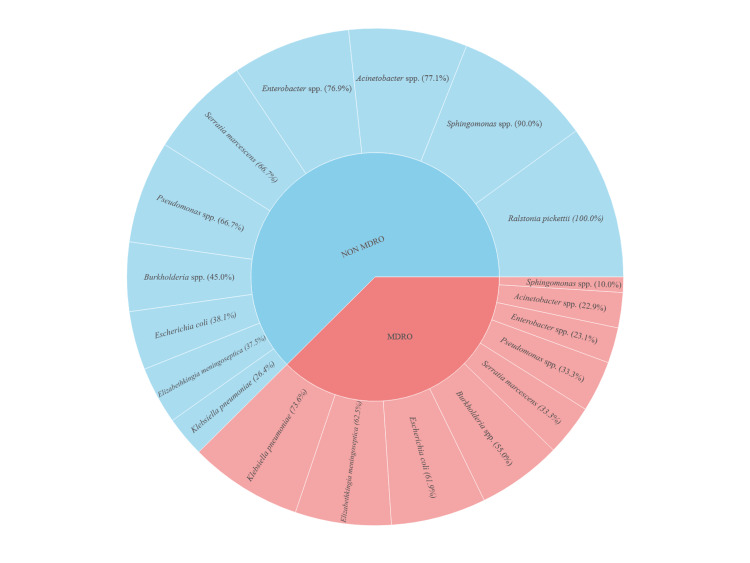
Distribution of MDR strains among total gram-negative bacilli in percentage (Sunburst) (N=201) MDR: multidrug resistant

Out of a total of 201 GNB, 92 MDR strains were detected. The highest percentage of MDR strains was observed among *K. pneumoniae* isolates (73.6%, 39/53), followed by *E. meningoseptica* (62.5%, 5/8) and *E. coli* (61.9%, 13/21). *Burkholderia* spp. also exhibited a notable MDR prevalence of 55.0% (11/20) (Figure 3).

**Figure 4:**
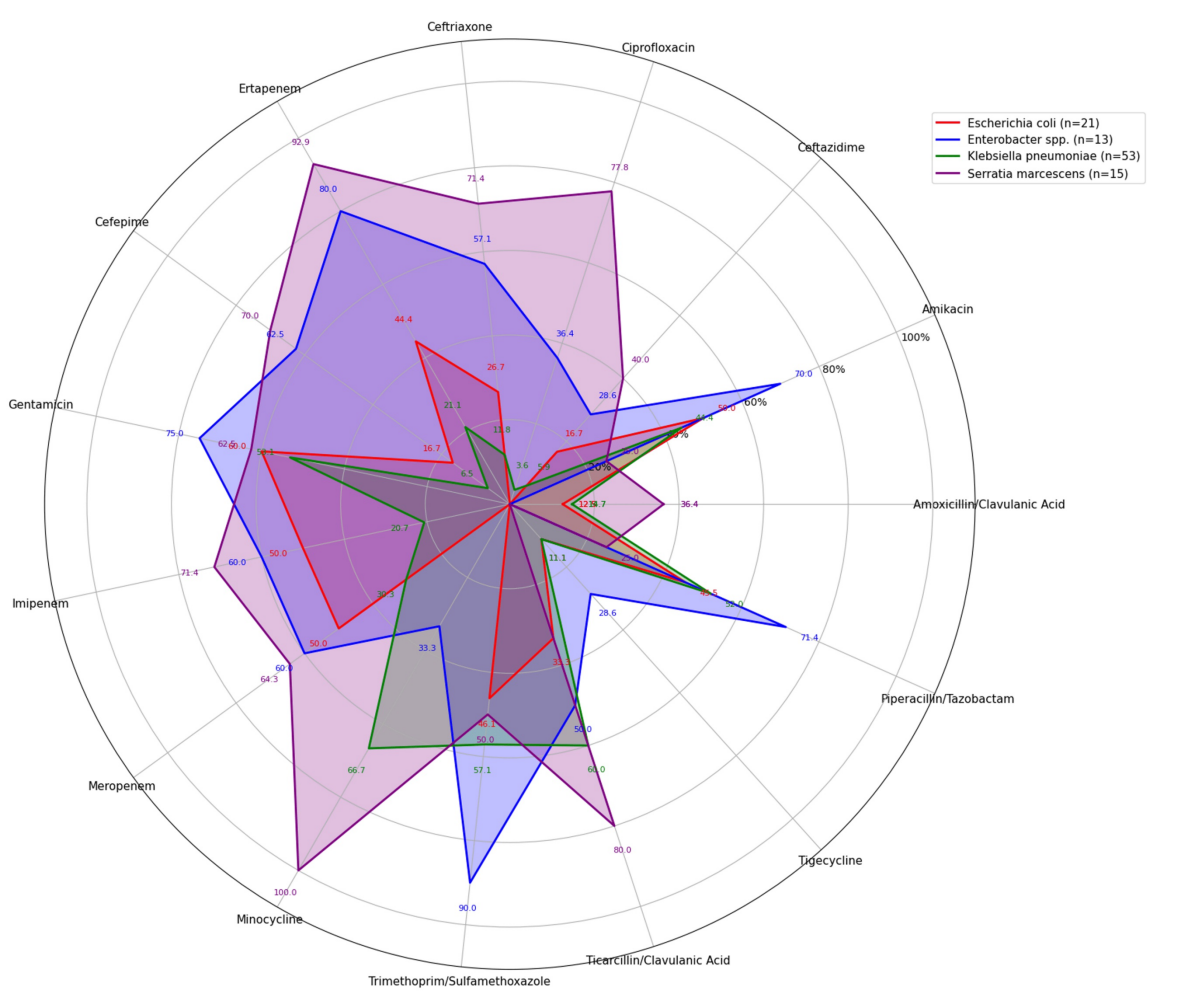
Antibiotic sensitivity pattern of gram-negative bacilli from Enterobacteriaceae family (Radar Chart)

Among GNB from the Enterobacteriaceae family, *K. pneumoniae* exhibited relatively low susceptibility to most of the antibiotics tested, with higher sensitivity observed for minocycline (66.67%), ticarcillin-clavulanic acid (60%), trimethoprim/sulfamethoxazole (57.64%), and gentamicin (53.13%). *E. coli* showed higher susceptibility to gentamicin (60%), amikacin (50%), imipenem (50%), and meropenem (50%). *S. marcescens* demonstrated good susceptibility to minocycline (100%), cefepime (92.86%), and ertapenem (71.43%). *Enterobacter *spp. demonstrated higher sensitivity to trimethoprim-sulfamethoxazole (90%), followed by ertapenem (80%), gentamicin (75%), and amikacin (70%). Overall, the findings highlighted varied resistance patterns among the species, underscoring the importance of regular antibiotic susceptibility testing to guide empirical treatment in neonatal sepsis (Figure 4).

**Figure 5:**
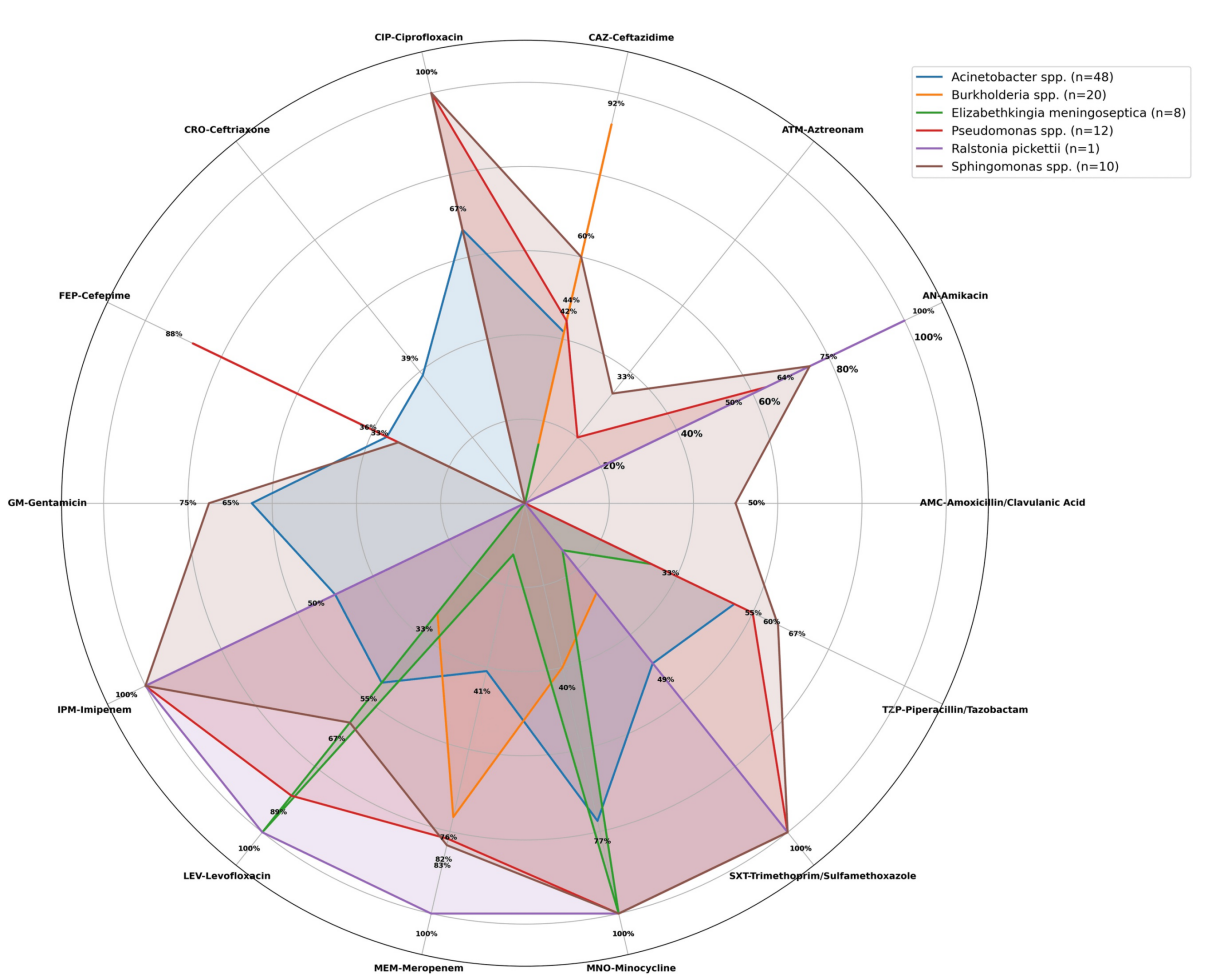
Antibiotic sensitivity pattern of non-fermenter gram-negative bacilli (Radar Chart)

Non-fermenter GNB isolated showed the following patterns of susceptibility: *Acinetobacter* spp. showed the highest susceptibility to minocycline (77.42%), followed by ciprofloxacin (66.67%) and gentamicin (64.86%). *Burkholderia* spp. exhibited limited susceptibility to most of the antibiotics, with the highest sensitivity observed for ceftazidime (92.31%), followed by meropenem (76.47%) and minocycline (40%). *Pseudomonas* spp. demonstrated excellent susceptibility to ciprofloxacin, imipenem (100% each), and levofloxacin (88.89%). *Sphingomonas* spp. showed high sensitivity to imipenem, meropenem, and trimethoprim/sulfamethoxazole (100% each). *E. meningoseptica *exhibited maximum sensitivity to levofloxacin (100%) and minocycline (100%), but displayed significant resistance to most other antibiotics, including beta-lactams and aminoglycosides. Only one isolate of *R. pickettii* showed complete susceptibility to aminoglycosides, carbapenems, and fluoroquinolones (100%), while being resistant to aztreonam and some beta-lactam combinations (Figure 5).

In this study, the association of various demographic parameters and outcomes with MDR GNB sepsis was evaluated. The comparison of clinical and demographic variables between MDR and non-MDR cases, like gender, gestational age, and mode of delivery, sepsis onset, birth place, or mortality, showed no statistically significant differences. However, birth weight differed significantly (p = 0.02), with the MDR group having higher proportions of LBW and VLBW neonates, while the non-MDR group had more ELBW infants. Preterm births were common in both groups, and mortality was higher in the non-MDR group, but neither of these differences was statistically significant. These findings highlight birth weight as a potential factor influencing MDR status (Table 2).

**Table 2:** Association of risk factors with MDR GNB sepsis (n = 201) * Chi Square Test   ** Fisher Exact test Df: degree of freedom;  MDR: multidrug resistant; GNB: gram-negative bacilli

Variables		MDR (n = 92), n (%)	Non-MDR (n = 109), n (%)	Total ( n=201), n (%)	Test of association	p-value
Gender	Female	33 (35.9%)	46 (42.2%)	79 (39.3%)	Chi-square value: 0.59 Df=1	0.36*
	Male	59 (64.1%)	63 (57.8%)	122 (60.7%)		
Gestational age at birth	Preterm	62 (67.4 %)	89 (81.7%)	151 (75.1%)	F- value: 0.45 Df=2	0.62**
	Term	30 (32.6%)	19 (17.4%)	49 (24.4%)		
	Post Term	0	1(0.9%)	1 (0.5%)		
Birth weight	>2500 g	20 (21.7%)	17 (15.6%)	37 (18.4%)	Chi-square value: 10.31 Df=3	0.02*
	Low birth weight	27 (29.3%)	16 (14.7%)	43 (21.4%)		
	Very Low birth weight	29 (31.6%)	43 (39.4%)	72 (35.8%)		
	Extremely Low birth weight	16 (17.4%)	33 (30.3%)	49 (24.4%)		
Mode of delivery	Lower segment caesarean section	47 (51.1%)	59 (54.1%)	106 (52.7%)	Chi-square value: 0.08 Df=1	0.66*
	Vaginal Delivery	45 (48.9%)	50 (45.9%)	95 (47.3%)		
Sepsis onset	Early onset Sepsis	35 (38%)	36 (33%)	71 (35.3%)	Chi-square value: 0.35 Df=1	0.55*
	Late onset sepsis	57 (62%)	73 (67%)	131 (64.7%)		
Birth Place	Inborn	38 (41.3%)	56 (51.4%)	94 (46.8%)	Chi-square value: 1.65 Df=1	0.15*
	Out born	54 (58.7%)	53 (48.6%)	107 (53.2%)		
Outcome	Death	14 (15.2%)	27 (24.8%)	41 (20.4%)	Chi-square value: 2.25 Df=1	0.13*
	Discharge	78 (84.8%)	82 (75.2%)	160 (79.6%)		

## Discussion

Neonatal sepsis is a major health concern in the first month of life, causing high morbidity and mortality. Timely diagnosis and careful antibiotic administration are vital to enhance treatment outcomes in neonates with sepsis. However, the indiscriminate use of broad-spectrum antibiotics has significantly contributed to the emergence of MDR strains [21]. The United States National Healthcare Safety Network (NHSN) has documented a rising prevalence of multidrug-resistant strains among MDR GNB, comprising *E. coli,* *K. pneumoniae*, and *Enterobacter* spp., with above 60% of cases attributed to *Acinetobacter *spp. [22]. Similarly, the European Antimicrobial Resistance Surveillance Network (EARS-Net) identified notable resistance trends among gram-negative pathogens, with *Acinetobacter* spp. showing the highest resistance rates, followed by *E. coli*, *K. pneumoniae *[23].

In the present study, the prevalence of GNB was found to be 47.4% among neonatal sepsis cases. This is consistent with findings of Moftian et al., who reported GNB prevalence as 53.6% in a NICU setting in Iran [6]. However, higher prevalence rates have been observed in other studies, such as Almohammady et al. (65.3%) in Egypt [24] and Gupta et al. (71.74%) in India [25]. The predominance of GNB in neonatal sepsis could be attributed to postnatal acquisition of environmental bacterial strains from the hospital setting, both of which have been well-documented in the study done by Kurma et al. [26]. As per the literature, observed variation in GNB prevalence is influenced by several factors such as geographical differences, infection control practices, and the level of neonatal intensive care provided. Furthermore, differences in study design, sample size, and laboratory diagnostic methods can also contribute to the variability in detection rates of GNB [27].

Furthermore, the present study showed a gender disparity among septic neonates, with a higher proportion of sepsis cases in male neonates (60.7%, n=122) compared to female neonates (39.3%, n=79). This male predominance is consistent with studies by Kurma et al. [26] and Doenhardt et al. [28], where male neonates with sepsis were found to be 51.4% and 61%, respectively. The higher proportion of male neonates in our study may be attributed to a greater admission rate of male infants compared to females in our setting.

In terms of perinatal characteristics, the majority of neonates were preterm (75.1%) and VLBW (35.8%). The predominance of prematurity and low birth weight among the study population aligns with known risk factors for neonatal infections. Preterm and low birth weight neonates have underdeveloped immune systems, reduced skin and mucosal barrier integrity, and often require invasive interventions, all of which increase their vulnerability to bacterial colonization and subsequent infection [29].

In our study, the most prevalent bacteria were Klebsiella pneumoniae (26.4%), followed by *Acinetobacter* spp. (23.9%), and *E. coli* (10.4%). The predominance of *K. pneumoniae* is consistent with findings from a study by Pataskar et al., conducted in central India, which reported a high prevalence rate of 36.7% [30]. This high prevalence of *Acinetobacter *spp. in our study surpasses the prevalence rate of 13.7% documented by Nazir et al., which indirectly gives evidence of the frequent existence of *Acinetobacter* spp. in our hospital setting [31]. We found the isolation rate of *E. coli* as 10.4% in our study, which is comparable to the 13.84% prevalence reported by Fang et al. in China [32]. Another study conducted in China by Xiao et al. also reported *E. coli* as a significant pathogen in neonatal sepsis, particularly in early-onset infections [33].

In the present study, *K. pneumoniae* was the most prevalent MDR bacterium (73.6%). This is higher than the rate of MDR *K. pneumoniae* isolates (54%) reported by the investigators of the Delhi Neonatal Infection Study (DeNIS) collaboration. Similarly, MDR *E. coli* isolates (61.9%) in our study exceed the rate of 38% as reported in the DeNIS collaboration study [34]. Another multicentric study conducted by Jain et al. across five district hospitals in India (2019-2021) reported 84.3% MDR *K. pneumoniae* isolates and 84.8% MDR strains in *E. coli* [35]. The higher isolation of MDR bacterial isolates contributes to increased mortality, prolonged hospitalization, and limited therapeutic options [36]. The emergence of resistance to reserve group antibiotics further demands the critical need for evidence-based, targeted antimicrobial strategies in neonatal care settings.

In our study, minocycline (66.67%), ticarcillin-clavulanic acid (60%), trimethoprim/sulfamethoxazole (57.64%), and gentamicin (53.13%) emerged as the most effective antibiotics for *K. pneumoniae*. Cefepime (6.45%) and imipenem (20.69%) demonstrated notably reduced activity against these isolates. In contrast, a previous study by Gupta et al. reported significantly the highest susceptibility of *K. pneumoniae* to imipenem (91%) followed by piperacillin-tazobactam (87%), emphasizing potential regional variations in antimicrobial resistance patterns [25]. Breakpoints for ticarcillin/clavulanic acid for *Klebsiella* spp. are available in CLSI guidelines, and the antibiotic combination is also available commercially in India. Due to a lack of clinical evidence regarding the use of certain antibiotics like minocycline and ticarcillin/clavulanic acid in neonates, these antibiotics may not be recommended for the treatment of sepsis cases, though they are tested in the present study. 

*E. coli *isolates from neonatal sepsis cases showed moderate susceptibility to aminoglycosides such as gentamicin (60%) and amikacin (50%), as well as to imipenem (50%) and meropenem (50%). These findings are in contrast to a study from China (Xiao et al. 2023), which reported higher susceptibility rates to amikacin (98%) and gentamicin (70%) among *E. coli *isolates [33]. Similarly, a study done by Dalal et al. in India (2021) documented sensitivity rates of 70% to amikacin and 64% to gentamicin in neonates with sepsis [37]. The observed differences may be influenced by underlying genetic mechanisms, such as the presence of antibiotic resistance genes due to mutations or by horizontal gene transfer from closely related strains, as well as variations in neonatal ICU infection control practices and antibiotic prophylaxis policies [38].

*Enterobacter* spp. in our study demonstrated the highest susceptibility, particularly to trimethoprim-sulfamethoxazole (90%), ertapenem (80%), gentamicin (75%), and amikacin (70%). These findings are partially aligned with those of Almohammady et al. (2023), who reported 67% susceptibility to amikacin, whereas 100% susceptibility was seen to levofloxacin [24]. However, our results diverge from those of Siddiqui et al., who found complete susceptibility to carbapenems (100%) and widespread resistance in other antibiotic classes [39]. Our results revealed the sensitivity of *S. marcescens* to minocycline as 100% and higher sensitivities to cefepime (92.86%) and ertapenem (71.43%).

In the present study, *Acinetobacter* spp. showed the highest susceptibility to minocycline (77.42%), followed by ciprofloxacin (66.67%) and gentamicin (64.86%). These findings contrast sharply with the results reported by the study conducted by Nazir et al. in northern India, who observed much lower susceptibility rates for minocycline (30.6%), ciprofloxacin (4.08%), and gentamicin (4.08%) [31]. *Acinetobacter *spp. are rapidly spreading, with rising resistance even to newer antimicrobials. Their ability to quickly acquire resistance and persist in hospital environments makes them potent causes of nosocomial outbreaks. The global rise of MDR *Acinetobacter* baumanii limits treatment options, highlighting the need for novel therapies.

*Burkholderia* spp. demonstrated high susceptibility to ceftazidime (92.31%) and meropenem (76.47%), findings that are consistent with those reported by Kar et al., who observed ceftazidime susceptibility of 87% among clinical isolates [40]. In our findings, *Pseudomonas* spp. showed higher sensitivity to ciprofloxacin (100%), imipenem(100%), meropenem (81.82%), and piperacillin-tazobactam (60%). In contrast, Dalal et al. reported higher susceptibility to meropenem (96%) and piperacillin-tazobactam (93%) but lower susceptibility to ciprofloxacin (47%) [37].

The susceptibility profile of *Sphingomonas* spp. revealed complete sensitivity to imipenem, meropenem, and trimethoprim/sulfamethoxazole. Another emerging bacteria, *E. meningoseptica*, was susceptible only to levofloxacin (100%) and minocycline (100%), consistent with findings by Anil et al., who reported that *E. meningoseptica* was susceptible to minocycline and levofloxacin while showing resistance to all other antibiotics tested [41].

MDR infections were significantly more common in neonates with low birth weight, particularly in the VLBW and ELBW groups (p = 0.02). MDR (58.7%) was more prevalent in outborn neonates, while higher mortality occurred in the non-MDR group (24.8%). Although variables such as gender, gestational age, mode of delivery, onset of sepsis, and mortality showed no significant association, these findings are consistent with those of Miranda et al. [42], who identified low birth weight and outborn status as risk factors, but differ from Zou et al. [43], who reported higher mortality with MDR infections, a discrepancy that may reflect differences in care quality and treatment practices. The higher MDR rate in LBW neonates may be explained by their immature immune systems, prolonged hospitalization, and increased use of invasive procedures, whereas the higher mortality in the non-MDR group could be due to severe underlying conditions or early-onset sepsis, where rapid disease progression leads to poor outcomes despite pathogen susceptibility.

Limitations

The present study has certain limitations. The retrospective study design may introduce selection and information biases. Due to a smaller number of CSF samples, the patients with CSF culture-positive cases were considered together with blood culture-positive neonates for analysis of antibiotic sensitivity and prevalence of MDR strains. Moreover, the lack of molecular characterization limits the ability to elucidate the specific mechanisms underlying antimicrobial resistance. Finally, the absence of long-term follow-up data restricts the assessment of the extended clinical impact of multidrug-resistant infections in neonates.

## Conclusions

GNB in neonatal sepsis has become a major concern, particularly in developing countries, because of the rising prevalence of MDR strains. This study highlights the burden of GNB in neonatal sepsis with the distribution of MDR strains among different species of GNB in a tertiary care health setting. The most predominant bacteria isolated were *K. pneumoniae, *followed by *Acinetobacter* spp., and *E. coli*. The maximum number of MDR strains was observed among *K. pneumoniae* isolates, followed by *E. meningoseptica* and *E. coli*. Species-specific susceptibility patterns were noted as *K. pneumoniae *showed higher sensitivity to trimethoprim/sulfamethoxazole and gentamicin; *E. coli* to gentamicin and imipenem;* Enterobacter *spp. to trimethoprim-sulfamethoxazole and ertapenem; Acinetobacter spp. to minocycline and ciprofloxacin; Pseudomonas spp. to ciprofloxacin, imipenem, and cefoperazone-sulbactam; *Burkholderia* spp. to ceftazidime and meropenem. LBW and VLBW neonates were significantly associated with sepsis caused by MDR GNB compared to ELBW neonates. These findings emphasize the importance of species-specific antimicrobial strategies and sustained resistance surveillance to guide effective management in NICUs through implementing appropriate antibiotic stewardship.

These findings highlight the urgent need for species-directed antimicrobial stewardship, routine resistance surveillance in neonatal intensive care units, and implementation of appropriate Infection Prevention and Control (IPC) practices. Appropriate empirical therapy based on local susceptibility trends is essential to improve clinical outcomes and reduce the burden of multidrug-resistant infections in this vulnerable population.
